# Role of DKxanthenes in *Myxococcus*–Nematode Interactions

**DOI:** 10.4014/jmb.2602.02003

**Published:** 2026-05-25

**Authors:** Hyesook Hyun, Gangmin Kim, Chaehyeon Park, Yujin Ka, Kyungyun Cho

**Affiliations:** Department of Biotechnology, Hoseo University, Asan 31499, Republic of Korea

**Keywords:** *Myxococcus*, DKxanthene, Myxobacteria, Nematode, *Caenorhabditis elegans*

## Abstract

Myxobacteria of the genus *Myxococcus* produce bioactive secondary metabolites known as DKxanthenes. When *Myxococcus stipitatus* DSM 14675^T^ was co-cultured with the nematode *Caenorhabditis elegans* under conditions that induce DKxanthene production, nematode activity and survival were strongly suppressed, and nematodes progressively disappeared. In contrast, under conditions in which DKxanthenes were not produced, or when DKxanthene-deficient mutant strains were used, nematodes proliferated. Similarly, myxobacteria of the genus *Corallococcus*, which belong to the same family as *Myxococcus* but lack the DKxanthene biosynthetic gene cluster, supported robust nematode growth during co-cultivation with *C. elegans*. Assays using partially purified DKxanthene extracts demonstrated that DKxanthenes inhibit the growth of *C. elegans*, although they do not cause immediate nematode death. Together, these findings indicate that DKxanthenes function as key antinematodal metabolites that suppress nematode growth and viability and contribute to nematode resistance in *Myxococcus* under nutrient-limited conditions.

## Introduction

Myxobacteria are soil-dwelling bacteria well known for producing a wide range of bioactive secondary metabolites [1-3]. Among these, DKxanthenes are yellow-pigmented compounds produced by the genera *Myxococcus* and *Stigmatella* [4, 5]. Structurally, DKxanthenes consist of long conjugated polyene chains synthesized via a hybrid polyketide synthase (PKS) and non-ribosomal peptide synthetase (NRPS) pathway. Variations in polyene chain length and specific side-chain modifications result in a family of derivatives with differing molecular weights; for instance, *Myxococcus stipitatus* DSM 14675^T^ is known to produce at least eight distinct variants [5]. Functionally, DKxanthenes exhibit both antioxidative and antifungal activities, and are specifically essential for developmental sporulation in *Myxococcus xanthus* [4, 5].

In nutrient-rich environments, myxobacteria proliferate by preying on other bacteria or utilizing organic matter present in their surroundings. When nutrients become limited, however, hundreds of thousands of cells aggregate at a single site to form fruiting bodies that rise above the surface. Within these structures, vegetative cells differentiate into spherical or oval-shaped spores known as myxospores, resulting in mature fruiting bodies [6]. Myxospores exhibit high resistance to environmental stresses such as heat, desiccation, and toxic chemicals. Upon nutrient replenishment, the spores germinate simultaneously and give rise to swarms of vegetative myxobacterial cells.

Myxobacteria are predatory bacteria that sustain themselves by killing and consuming other microorganisms [7]. They display collective behavior and secrete a variety of toxic secondary metabolites and hydrolytic enzymes that effectively kill and lyse their prey. These compounds are secreted into the extracellular medium or delivered to prey cells via outer membrane vesicles (OMVs) [8]. In addition, recent studies have demonstrated that myxobacteria can kill prey through direct cell-to-cell contact by injecting toxic compounds [9]. Although myxobacteria were traditionally considered predators of other bacteria, recent findings indicate that some species can also prey on small eukaryotes, including fungi [10], oomycetes [11], and ciliates [12].

Myxobacteria primarily inhabit soil ecosystems, where they compete with a wide diversity of microorganisms. Depending on soil type, 1 g of soil from a depth of 0–15 cm typically contains approximately 10^8^–10^9^ bacterial cells, 10^5^–10^6^ fungal cells, 10^4^–10^5^ algal cells, 10^3^–10^4^ protozoa, and 10^2^–10^3^ nematodes [13]. Among these, bacterivorous nematodes actively migrate through soil matrices and feed on bacterial populations, suggesting that myxobacteria may require defensive strategies to resist nematode predation.

*M. xanthus* undergoes phase variation between yellow and tan morphotypes, which differ in several phenotypic traits, including secondary metabolite production, aggregation behavior, swarming motility, and fruiting body formation [14-16]. In a separate study, Dahl *et al*. reported that the yellow variant exhibits enhanced biofilm formation, increased sensitivity to lysozyme, and greater resistance to ingestion by the bacterivorous nematode *Caenorhabditis elegans* compared with the tan variant [17].

In our previous study, we identified a DKxanthene biosynthetic gene cluster from *M. stipitatus* DSM 14675^T^ and constructed mutants defective in DKxanthene production [3]. Because DKxanthene production is one of several phenotypic differences between the yellow and tan morphotypes, the present study aimed to investigate the role of DKxanthene production in *Myxococcus*–nematode interactions.

## Materials and Methods

### Strains, Media, Culture Conditions

The strains *M. stipitatus* DSM 14675^T^ [18], *M. xanthus* DSM 16526^T^, and *Corallococcus coralloides* DSM 2259^T^ were obtained from the Deutsche Sammlung von Mikroorganismen und Zellkulturen GmbH (DSMZ). *M. xanthus* DK1622 was obtained from the laboratory of Prof. David R. Zusman at the University of California, Berkeley. *C. elegans* N2 and *Escherichia coli* OP50-1 were obtained from the laboratory of Prof. Junho Lee at Seoul National University. *M. stipitatus* KYC2006 and *C. coralloides* KYC2213 are strains in the lab collection. Mutant strains KYC595, KYC617, and KYC644, derived from *M. stipitatus* DSM 14675^T^ with inactivated biosynthetic genes for melithiazols, DKxanthenes, and phenalamides respectively, were constructed in a previous study [5, 19, 20].

Casitone-yeast extract (CYE) medium [21] was used for general cultivation of myxobacteria. Casitone-yeast extract-starch (CYS) medium [22] was used for DKxanthene production, and soytone-yeast extract (SYE) medium [23] was used for dispersed growth. Nematode growth medium (NGM) [24] containing *E. coli* OP50-1 was used to culture nematodes [25]. Myxobacteria were incubated at 32°C, *E. coli* at 37°C, and nematodes at 20°C.

### Analysis of Antinematodal Activity of Myxobacteria

Myxobacterial strains were cultured in 250-mL Erlenmeyer flasks containing 50 mL of SYE medium with agitation at 150 rpm and 32°C. Cultures reached an optical density at 600 nm (OD_600_) of 1.0 within 24-36 h. A 100-μL aliquot of the myxobacterial culture was spread onto CYS or CYE agar plates and incubated at 32°C for 5 days. A 10-μL nematode suspension, containing approximately 50 individuals ranging from L1 to adult stages, was then placed in the center of the agar plates pre-inoculated with myxobacteria, followed by incubation at 20°C for 5 days. To transfer the nematode suspension, wide-bore micropipette tips (prepared by cutting the tips to enlarge the opening) were used. To prevent dehydration, all plates were sealed with plastic wrap during incubation. The surviving nematodes were then observed with a SMZ1000 stereomicroscope (Nikon, Japan) and counted.

### Quantitative Analysis of DKxanthene Production

Myxobacteria were cultured in SYE medium until OD_600_ reached 1.0. Then, 100 μL of the culture was inoculated into 50 mL of liquid medium or plated on an agar plate, incubated for 5 days, and subsequently harvested. The harvested cells were weighed using an analytical balance, with wet weights ranging from 21.5 to 1,472.7 mg (Supplementary Table S1). The cells were extracted in a 15-mL conical tube three times with 3.3 mL of methanol each (total volume 9.9 mL). In each cycle, the mixture was vortexed for 10 s at maximum speed and centrifuged at 4,000 rpm (approximately 2,600 × g) for 5 min. The supernatants were then pooled, and additional methanol was added to reach a final volume of 10 mL. The extracts were analyzed using an HPLC system (1260 VL Infinity Series; Agilent, USA) equipped with a Zorbax SB-C18 column (4.6 × 150 mm, 5 μm; Agilent). Water containing 0.1% trifluoroacetic acid (TFA) and acetonitrile containing 0.1% TFA were used as mobile phases A and B, respectively. Gradient elution was performed at a flow rate of 1 mL/min as follows: 0–10 min, 40–50% B (linear gradient); 10–13 min, 50–100% B (linear gradient); 13–16 min, 100% B (isocratic); and 16–20 min, 40% B (isocratic). DKxanthene derivatives exhibit characteristic absorption spectra with a maximum at 410 nm. Therefore, DKxanthene peaks were identified by monitoring the eluents with a photodiode array (PDA) detector and verifying the absorption spectrum of each peak. For quantification, chromatograms recorded at 410 nm were used. The relative production levels of DKxanthenes were calculated by summing the peak areas of all DKxanthene derivatives, dividing the total by the cell weight used for extraction (Supplementary Table S1), and then normalizing the value to 1 for the production level in CYS liquid medium.

### Partial Purification of DKxanthenes

The entire content (cells and agar) from 100 CYS agar plates for 5 days was collected, chopped into small pieces, and extracted with methanol. The extract was then evaporated to dryness using a rotary evaporator. The resulting residue was partitioned between in a 1:1 mixture of water and ethyl acetate. The ethyl acetate layer was then collected and concentrated to dryness once more using a rotary evaporator. The resulting material was dissolved in 100% methanol and loaded onto an XK 16/70 column (Φ16 × 700 mm, GE Healthcare, USA) packed with Sephadex LH-20 resin (GE Healthcare) for gel permeation chromatography. DKxanthene fractions were then identified by HPLC analysis, recovered, dried, and re-dissolved in 100% methanol at a concentration of 100 mg/mL. The purity of the recovered material was estimated based on the peak area at 254 nm. HPLC analysis revealed that DKxanthene peaks accounted for approximately 10.1% of the total peak area, although the absolute purity was not further determined.

**Analysis of Antinematodal Activity of DKxanthenes**

Each well of a 24-well plate was filled with 0.2 mL of NGM liquid medium and 60 μL of *E. coli* OP50-1 suspension (5 × 10^8^ cells). Subsequently, 10 μL of nematode suspension, containing approximately 50 individuals ranging from L1 to adult stages, was added using wide-bore micropipette tips. The partially purified extract of DKxanthenes was serially diluted with methanol, and then 2 μL of each solution was added to obtain final concentrations of 0, 46, 92, 184, 368, and 735 μg/mL. After incubation at 20°C for 5 days in a plastic box with a few ventilation holes containing damp sponges, the number of nematodes was counted with a CKX31 inverted microscope (Olympus, Japan).

### Statistical Analysis

All experiments were performed in triplicate. Results are expressed as the mean ± standard deviation of three independent experiments. Statistical analyses were conducted using GraphPad Prism (GraphPad Software, USA).

## Results

### Media-Dependent DKxanthene Production in *M. stipitatus* DSM 14675

**Fig. 1:**
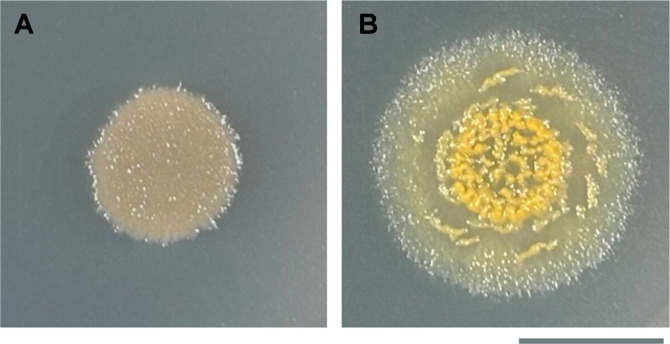
Morphology and pigmentation of *Myxococcus stipitatus* DSM 14675^T^ on CYE and CYS media. A 20-μl spot containing 1 × 10^7^ cells of *M. stipitatus* DSM 14675 was placed on CYE (**A**) and CYS (**B**) agar plates and incubated for 5 days. In panel B, yellow structures at the center of the spot are fruiting bodies. Bar: 10 mm.

*M. stipitatus* DSM 14675^T^ produced yellow pigments consistent with DKxanthenes when cultured on CYS agar medium, but not on CYE agar

medium (Fig. 1). The nutrient-rich CYE agar medium supported vegetative growth, with cells remaining in a rod-shaped state and failing to form fruiting bodies (Fig. 1A). In contrast, the relatively nutrient-limited CYS agar medium supported myxobacterial growth followed by the formation of yellow multicellular fruiting bodies upon nutrient depletion (Fig. 1B). These observations suggested that DKxanthene production in *M. stipitatus* DSM 14675^T^ is influenced by growth conditions and physiological state.

**Fig. 2:**
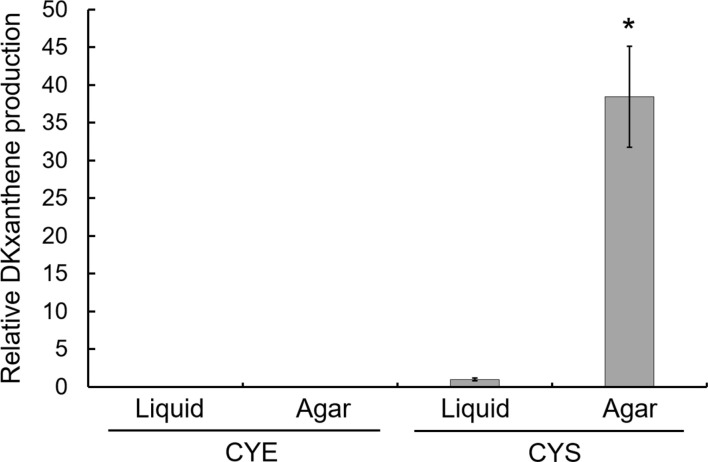
Media-dependent DKxanthene production in *M. stipitatus* DSM 14675^T^. Cells grown on different media were extracted with methanol, and DKxanthene production was quantified using HPLC. Relative DKxanthene production was determined by dividing the total peak area of all DKxanthene derivatives by the cell weight used for extraction (Supplementary Table S1) and normalizing the value to that obtained in CYS liquid medium (set as 1). *p < 0.05 compared to DKxanthene production on CYS liquid medium.

To examine this possibility, strain DSM 14675^T^ was cultured for 5 days in CYE liquid, CYE agar, CYS liquid, and CYS agar media. Cells were harvested and extracted with 100% methanol, and DKxanthene production was quantified by HPLC analysis. No DKxanthenes were detected in cells grown in either CYE liquid or agar media (Fig. 2). In contrast, cells grown in CYS liquid medium produced low levels of DKxanthenes, whereas cells grown on CYS agar medium produced 38.5-fold higher amounts than those grown in CYS liquid medium (Fig. 2). Together, these results indicate that DKxanthene production in *M. stipitatus* DSM 14675^T^ is suppressed under nutrient-rich conditions but induced under nutrient-limited conditions, with maximal production occurring under conditions that promote fruiting body formation. Notably, this production was not restricted to optimal growth temperatures; HPLC analysis confirmed that strain DSM 14675^T^ successfully produced DKxanthenes even at 20°C on CYS plates, achieving 13.5% of the yield observed at 32°C. In contrast, no production was detected on CYE plates, regardless of the incubation temperature.

### Media-Dependent Antinematodal Activity of *M. stipitatus* DSM 14675

To investigate the role of DKxanthenes in interactions between *M. stipitatus* and nematodes, differences in DKxanthene production under distinct culture conditions were exploited. *M. stipitatus* DSM 14675^T^ was spread onto CYE and CYS agar plates and cultured for 5 days, after which approximately 100 nematodes were placed on each plate. The plates were incubated, and nematode behavior was monitored every 24 h under a stereomicroscope.

**Fig. 3:**
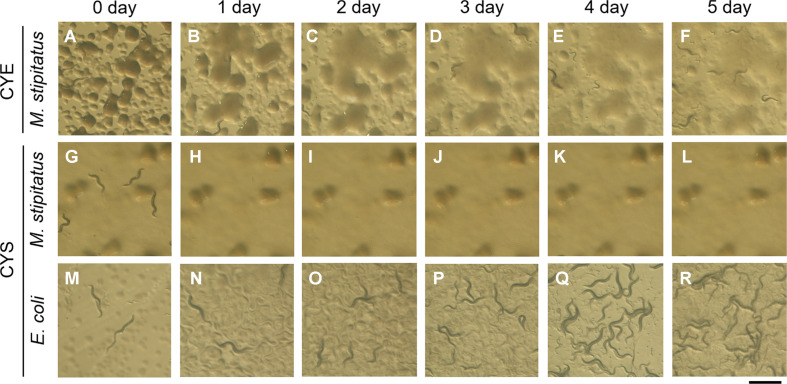
Co-cultivation of *M. stipitatus* DSM 14675^T^ with nematodes. Approximately 100 *Caenorhabditis elegans* were placed on a CYE or CYS agar plate inoculated with *M. stipitatus* DSM 14675 or *E. coli* OP50-1 and incubated at 20°C. In the CYS medium, due to nutrient depletion, the myxobacteria form multicellular fruiting bodies, as observed in panels G–L. In contrast, in the CYE medium, no fruiting bodies are formed because the medium is nutrient-rich, and therefore, such structures are not observed in panels A–F. Scale bar: 1 mm.

On CYE agar plates inoculated with *M. stipitatus*, nematodes at various developmental stages—ranging from eggs to adults—remained actively motile throughout the observation period. Their population increased from the initial inoculum to approximately 580 individuals within 5 days, indicating growth by feeding on myxobacterial cells (Fig. 3A–3F). On CYS agar plates inoculated with *E. coli*, nematodes remained actively motile and their population increased to approximately 20,000 individuals after 5 days (Fig. 3M–3R).

In contrast, on CYS agar plates inoculated with *M. stipitatus*, some adult nematodes ceased movement within 1 day, gradually became transparent, and eventually disappeared. By day 3, only a few small, apparently dead nematodes were observed on CYS plates (Fig. 3J). This behavior differed markedly from that observed on CYE plates, suggesting that *M. stipitatus* grown on CYS medium adversely affected nematode survival. Given that DKxanthenes (yellow pigments) are not produced under CYE conditions but are abundantly produced under CYS conditions, these observations strongly suggest that DKxanthenes are likely involved in the nematicidal activity.

### Antinematodal Activity of DKxanthenes

*M. stipitatus* DSM 14675^T^ is known to produce several antifungal secondary metabolites, including DKxanthenes, melithiazols, and phenalamides. Melithiazols and phenalamides inhibit mitochondrial electron transport [19, 20], suggesting they may possess antinematodal activity similar to DKxanthenes. To determine if these compounds contribute to nematode growth inhibition and lethality, we evaluated the inhibitory effects of mutant strains KYC595 (melithiazol-deficient), KYC617 (DKxanthene-deficient), and KYC644 (phenalamide-deficient), using the wild-type strain DSM 14675 as a control. These strains were cultured on CYS solid medium for 5 days. Subsequently, approximately 50 nematodes were placed at the center of each plate and incubated for an additional 5 days.

On plates seeded with strains DSM 14675^T^, KYC595, or KYC644, neither live nor dead nematodes were observed. In contrast, an average of 296 living nematodes were observed on plates seeded with strain KYC617, which does not produce DKxanthenes (Table 1). Taken together, these results indicate that DKxanthenes are primary contributors to nematode mortality, whereas melithiazols and phenalamides appear to have a negligible effect.

**Fig. 4:**
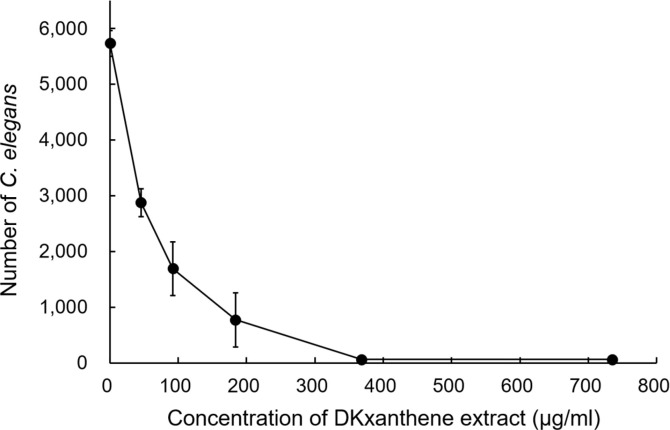
Antinematode activity of DKxanthenes. Approximately 50 *C. elegans* were co-incubated with *E. coli* in NGM liquid medium containing partially purified DKxanthene extract in 24-well plates for 5 days, and nematode numbers were counted.

To further assess whether DKxanthenes directly affect nematode viability, DKxanthenes were partially purified from *M. stipitatus* DSM 14675^T^ and added to nematode growth medium (NGM) at final concentrations of 0, 46, 92, 184, 368, and 735 μg/mL. Approximately 50 nematodes were added to each well of a 24-well plate, and nematode activity was monitored under an inverted microscope during incubation (Fig. 4).

After 1 day, sluggish movement was observed in some nematodes in wells containing 368 and 735 μg/mL of the DKxanthene extract. After 3 days, most nematodes in these wells exhibited no movement, and their numbers did not increase even after 5 days of incubation, indicating complete inhibition of growth (Fig. 4). In contrast, nematodes in the methanol control wells (0 μg/mL DKxanthene extract) remained highly active, and their population increased from approximately 50 to an average of 5,733 individuals after 5 days, corresponding to an approximately 115-fold increase. Under the same conditions, nematode numbers in wells containing 46, 92, and 184 μg/mL DKxanthene extract increased to average values of 2,873, 1,693, and 773 individuals, respectively, demonstrating a clear concentration-dependent inhibition of nematode growth (Fig. 4). Collectively, these findings demonstrate that DKxanthenes inhibit nematode activity and growth.

### Antinematodal Activities of Diverse *Corallococcus* and *Myxococcus* Strains

Genomic analyses revealed that all species within the genus *Myxococcus* possess the DKxanthene biosynthetic gene cluster (BGC), whereas species of the closely related genus *Corallococcus* lack this cluster. To investigate how the presence or absence of the DKxanthene BGC influences interactions with nematodes, strains of *Myxococcus* and *Corallococcus* were spread on CYS agar medium and incubated for 5 days. Approximately 50 nematodes were then placed on each plate, followed by an additional 5 days of incubation.

On plates inoculated with *Corallococcus coralloides* DSM 2259^T^, an average of 9,365 nematodes were observed, whereas plates inoculated with *C. coralloides* KYC2213 contained an average of 1,270 nematodes (Table 2). In contrast, fewer than 10 nematodes were detected on plates inoculated with *M. stipitatus* or *M. xanthus* strains. Together, these results suggest that *Corallococcus* strains lacking the DKxanthene BGC are poorly protected against nematodes, whereas *Myxococcus* species possessing this cluster exhibit strong resistance to nematode proliferation.

## Discussion

Myxobacteria frequently encounter diverse eukaryotic organisms in soil ecosystems, including bacterivorous nematodes. *C. elegans* feeds on a wide range of bacteria, including myxobacteria [17, 26]. As predatory bacteria, myxobacteria typically acquire nutrients by killing and decomposing other microorganisms through the synergistic action of toxic secondary metabolites and hydrolytic enzymes in close proximity to their prey [7, 8]. These activities enable efficient lysis of prey cells and subsequent nutrient acquisition.

In this study, we show that interactions between myxobacteria and *C. elegans* are strongly modulated by growth conditions and the associated secondary metabolite profiles. Nutrient limitation triggers myxobacteria to form multicellular fruiting bodies composed of metabolically dormant spores, which may be particularly vulnerable to grazing by bacterivorous nematodes. It is therefore likely that myxobacteria employ protective strategies to safeguard these developmental structures. Previous studies have shown that fruiting bodies are surrounded by metabolically active, rod-shaped peripheral cells [27]. Our results suggest that DKxanthene-producing peripheral rods inhibit nematode activity and survival, thereby protecting the spores within the fruiting bodies from nematode attack.

Assays using partially purified DKxanthene extracts demonstrated that DKxanthenes inhibit the growth of nematodes. However, DKxanthenes alone do not cause immediate nematode death. Even at high concentrations (≥368 μg/mL), nematodes survived for more than one day, although their movement and growth were strongly inhibited. Moreover, in extract-based assays, dead nematodes remained visible after prolonged incubation. In contrast, when nematodes were exposed to DKxanthene-producing myxobacterial cells on CYS agar, nematode remains were rarely observed and nematodes progressively disappeared. This distinction indicates that DKxanthenes account for nematode killing or growth inhibition, whereas additional myxobacteria-associated processes—such as extracellular enzymes or close physical interactions—likely promote rapid degradation or clearance of nematode remains.

DKxanthenes are nearly insoluble in water; however, Berleman *et al*. reported that *M. xanthus* outer membrane vesicles (OMVs) contain these compounds [28]. It is therefore suggested that myxobacteria deliver DKxanthenes to nematodes via OMVs in aqueous environments. Consequently, it is plausible that isolated DKxanthenes exhibit lower nematicidal activity than their counterparts encapsulated within OMVs.

An additional insight arises from experiments using the DKxanthene-deficient mutant KYC617. Although strain KYC617 does not produce DKxanthenes, nematodes population on CYS agar plates inoculated with this strain reached only about 296 individuals after 5 days—far fewer than the ~20,000 individuals observed on the DSM 14675^T^–*E. coli* control plates. This result indicates that strain KYC617 cells grown under CYS conditions do not serve as a suitable food source for nematodes, despite the absence of DKxanthene production. Therefore, it is likely that *M. stipitatus* produces additional nematode-resistant factors under nutrient-limited conditions, independent of DKxanthenes. Such factors may further reduce nematode feeding efficiency or survival and act synergistically with DKxanthenes to enhance protection of fruiting bodies. Similarly, in *C. coralloides* strains lacking DKxanthene production, strain KYC2213 exhibited relatively stronger resistance to nematodes than strain DSM 2259^T^, further supporting the existence of as-yet unidentified nematode-resistant factors.

Comparative genomic analyses further support the ecological significance of DKxanthene biosynthesis as a key determinant of nematode resistance in *Myxococcus*. Myxobacteria of the genus *Corallococcus*, which belong to the same family as *Myxococcus* but lack the DKxanthene BGC, sustained robust nematode growth during co-cultivation with *C. elegans*. While all *Myxococcus* species consistently possess the DKxanthene BGC, their overall secondary metabolite profiles often vary significantly. For instance, the genome of *M. stipitatus* DSM 14675^T^ contains melithiazol and phenalamide BGCs, which are absent in the *M. xanthus* DK1622 genome. Despite these differences, both strains exhibit strong nematicidal activity. This reinforces the role of DKxanthenes as the primary conserved defensive metabolites across the genus.

However, it should be noted that these experiments were conducted under controlled laboratory conditions. Consequently, while our findings establish a clear mechanism, further in situ studies are warranted to validate whether *Myxococcus* strains effectively produce DKxanthenes and suppresse nematode populations under more diverse and fluctuating natural environments.

Nematodes are ubiquitous in soil and aquatic environments, where they play important roles as decomposers and regulators of microbial communities. However, certain nematode species are serious agricultural pests that cause substantial crop losses [29]. Although chemical nematicides remain the most effective control agents, their use raises concerns regarding environmental contamination and toxicity to non-target organisms. Biological control strategies using microorganisms have been explored [30], but their efficacy is often limited. In this context, our findings highlight DKxanthene-producing myxobacteria—and potentially additional antinematodal factors induced under nutrient limitation—as promising resources for environmentally friendly nematode control in agricultural and ecological settings.

## Supplemental Materials

Supplementary data for this paper are available on-line only at http://jmb.or.kr.



## Figures and Tables

**Table 1:** Effects of inactivation of secondary metabolite biosynthetic genes on the antinematodal activity of *Myxococcus stipitatus*.

*Myxococcus stipitatus*	Genotype (phenotype)	Number of survived *Caenorhabditis elegans*
DSM 14675^T^	Wild type	1±2
KYC595	*melB*::pSH123 (melithiazol^−^)	0±0
KYC617	*dkxE*::pHS221 (DKxanthene^−^)	296±33
KYC644	*MYSTI_04326*::pSH109 (phenalamide^−^)	0±0

Approximately 50 nematodes were placed on CYS plates spread with myxobacteria and incubated for 5 days, after which nematode numbers were counted.

**Table 2:** Antinematode effects of *Corallococcus* and *Myxococcus* strains.

Species	Strain	Number of survived *C. elegans*
*Corallococcus coralloides*	DSM 2259^T^	9,365±5,706
*Corallococcus coralloides*	KYC2213	1,270±755
*Myxococcus stipitatus*	DSM 14675^T^	1±2
*Myxococcus stipitatus*	KYC2006	4±4
*Myxococcus xanthus*	DK1622	3±6
*Escherichia coli*	OP50-1	19,967±3,983

Approximately 50 nematodes were placed on CYS plates spread with myxobacteria and incubated for 5 days, after which nematode numbers were counted.
