# Supplementary material for: Role of DKxanthenes in Myxococcus–Nematode Interactions
Source: J Microbiol Biotechnol. 2026 May 25;36:e2602003. doi: 10.4014/jmb.2602.02003 (PMC13218925; doi:10.4014/jmb.2602.02003)
Supplement: Supplementary file 1 [file jmb-36-e2602003-supple.pdf]

Supplementary Table S1. Raw data used for Fig. 2 showing media-dependent DKxanthene production in *Myxococcus stipitatus* DSM 14675^{T}.

Medium	Replicate	Wet cell weight (mg)	HPLC peak area	Peak area / wet cell weight	Mean	SD
CYE broth	1	396.0	0.0	0.00	0.000	0.00
	2	680.9	0.0	0.00		
	3	992.2	0.0	0.00		
CYE plate	1	211.8	0.0	0.00	0.000	0.00
	2	174.0	0.0	0.00		
	3	184.8	0.0	0.00		
CYS broth	1	1,472.7	568.3	0.39	0.418	0.08
	2	1,439.0	522.7	0.36		
	3	1,325.2	669.2	0.50		
CYS plate	1	21.5	292.1	13.59	16.076	2.79
	2	69.1	1,319.1	19.09		
	3	66.2	1,029.5	15.55		

Wet cell weight represents the harvested biomass used for extraction. Peak areas correspond to the DKxanthene peaks detected by HPLC.
